# Detection of developmental dysplasia of the hip in X-ray images using deep transfer learning

**DOI:** 10.1186/s12911-022-01957-9

**Published:** 2022-08-13

**Authors:** Mohammad Fraiwan, Noran Al-Kofahi, Ali Ibnian, Omar Hanatleh

**Affiliations:** 1 Department of Computer Engineering, Jordan University of Science and Technology, Irbid, Jordan; 2 Department of Internal Medicine, Jordan University of Science and Technology, Irbid, Jordan

**Keywords:** DDH, Deep learning, X-ray, Diagnosis, Screening, Machine learning, Artificial intelligence

## Abstract

**Background:**

Developmental dysplasia of the hip (DDH) is a relatively common disorder in newborns, with a reported prevalence of 1–5 per 1000 births. It can lead to developmental abnormalities in terms of mechanical difficulties and a displacement of the joint (i.e., subluxation or dysplasia). An early diagnosis in the first few months from birth can drastically improve healing, render surgical intervention unnecessary and reduce bracing time. A pelvic X-ray inspection represents the gold standard for DDH diagnosis. Recent advances in deep learning artificial intelligence have enabled the use of many image-based medical decision-making applications. The present study employs deep transfer learning in detecting DDH in pelvic X-ray images without the need for explicit measurements.

**Methods:**

Pelvic anteroposterior X-ray images from 354 subjects (120 DDH and 234 normal) were collected locally at two hospitals in northern Jordan. A system that accepts these images as input and classifies them as DDH or normal was developed using thirteen deep transfer learning models. Various performance metrics were evaluated in addition to the overfitting/underfitting behavior and the training times.

**Results:**

The highest mean DDH detection accuracy was 96.3% achieved using the DarkNet53 model, although other models achieved comparable results. A common theme across all the models was the extremely high sensitivity (i.e., recall) value at the expense of specificity. The F1 score, precision, recall and specificity for DarkNet53 were 95%, 90.6%, 100% and 94.3%, respectively.

**Conclusions:**

Our automated method appears to be a highly accurate DDH screening and diagnosis method. Moreover, the performance evaluation shows that it is possible to further improve the system by expanding the dataset to include more X-ray images.

## Background

Developmental dysplasia of the hip (DDH) is a relatively common disorder in newborns with a reported prevalence of 1–5 per 1000 births [1], and recent studies indicate that there is a possibly higher incidence rate [2]. Hip dysplasia is a deformity that leads to structural instability and capsular laxity. DDH can result in developmental abnormalities in terms of mechanical difficulties, a displacement of the joint (i.e., subluxation or dysplasia), additionally, malformed growth and can eventually cause arthritis if left untreated [3]. Early diagnosis in the first few months from birth can drastically improve healing, render surgical intervention unnecessary and reduce the bracing time [4]. Pelvic X-ray inspection represents the gold standard for DDH diagnosis [5].

**Fig. 1:**
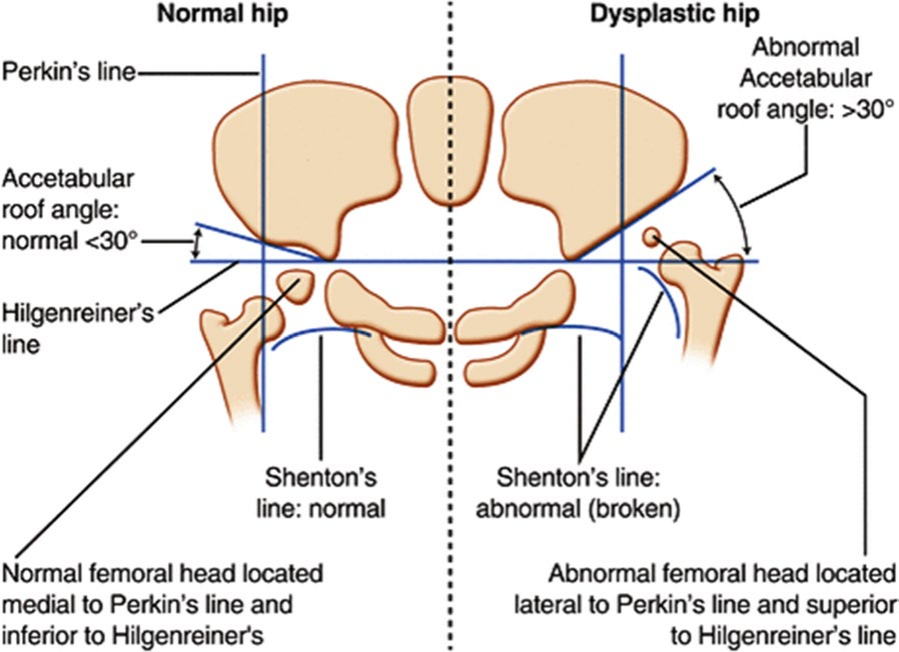
The pediatric radiographic parameters used to assess normal and dislocated hip [7]

Accurate diagnosis of DDH requires specialist knowledge of hip development and the alignment of the acetabulum and femoral head. Several possible acetabulum deformities may exist. Moreover, the treatment effectiveness and accuracy may require follow-up imaging and an inspection of the hip [4]. Figure 1 shows some of the most common pediatric pelvic parameters used to assess hip X-ray images as either normal or DDH. In pediatrics, a hip is judged as dysplastic based on the acetabular angle being greater than 30°for a newborn, a broken Shenton’s line, or an abnormal location of the femoral head (if ossified and visible) [6].

Recent advances in deep learning artificial intelligence have enabled many image-based medical decision-making applications. Deep learning is concerned with building neural networks with a number of layers that far exceed the traditional three (i.e., input, output and hidden). The late part of the last decade has witnessed a resurgence and proliferation of deep learning-based applications powered by the computational prowess of graphical processing units (GPUs) [8]. Convolutional neural networks (CNNs) are one of the most commonly used deep learning networks in the research literature. It is characterized by a series of convolution, pooling and rectified linear unit (ReLU) layers that conclude in a fully connected layer that combines the various features discovered by the subsequent layers. CNNs have been found to be useful for discovering features in images of various shapes from a wide range of medical specialties regardless of any scale, rotation, or translation [9]. Some application examples include eardrum otoendoscopic images [10], lung X-ray images [11], and images of white blood cells [12].

Regarding skeletal and bone-related diseases, deep learning has been used in many studies that detect bone diseases (e.g., cancers, arthritis, etc.) or deformities. Liu et al. [13] used deep learning to build a diagnostic CNN model of bone metastasis on bone scintigrams. Their approach works by performing a region classification using Resnet34, which is followed by a segmentation using U-net. A segmentation map is fed into the CNN model, which generates the diagnosis report. Jakaite et al. [14] employed a deep learning strategy of the group method of data handling (GMDH) to detect osteoarthritis. Fraiwan et al. [15] used deep transfer learning for the classification of vertebral X-ray images into spondylolisthesis, scoliosis, or normal. Other bone-related applications include bone age assessment [16–18], bone mineral density prediction [19] and fracture detection [20].

Given these recent advances, the DDH identification literature has been lagging behind in employing such powerful tools. Most of the related works still employ image processing techniques to automatically detect pelvic landmarks, delineate important lines (e.g., Hilgenreiner’s), and/or estimate certain angle measurements (e.g., the acetabular angle). To this end, Xu et al. [21] used a multitask hourglass network to detect six hip landmarks and the age of the femoral head. They achieved an average pixel error of 4.64, however, this type of metric may be susceptible to changes in the scale of the image. Al-Bashir et al. [22] detected important features in pelvic X-ray images using Canny edge detection. Then, the bread first search was used to locate possible femur head center locations. After that, the Hough transform was employed to find possible edges, and the best candidate was chosen based on eigenvalues and covariance matrices. Finally, the acetabular and center edge angles were estimated. They reported an accuracy range of 78.4-85.4%. Toward a similar result, Xu et al. [23] used object detection using mask-region based convolutional neural networks (Mask-RCNN) followed by a high resolution network (HRNet) for landmark detection and extraction. After that, the ResNet50 model was used for classification. Sahin et al. [24] constructed a model template image based on expert opinion and used comparisons with that template to find the best fitting (i.e., diagnosis). However, this type of method depends on the quality, scale, rotation, and shape of the pelvic image being similar to the template. Liu et al. [25] used local morphological and global structural features of the pelvis along with pyramid nonlocal UNet (PN-UNet) and reported an AA measurement average accuracy of 90.1% (right) and 89.8% (left).

All of the aforementioned literature follow a common traditional theme of attempting to estimate the location of the pelvic landmarks and the associated radiographic parameters. However, such approaches do not measure the impact of the measurement error on the diagnosis (e.g., a 3°error could be the same as 5°if both result in the same wrong diagnosis). Moreover, the multiple steps in each method may lead to compounded errors. In addition, the images may require explicit processing. In this work, we utilize advances in deep learning to automatically diagnose DDH from X-ray images in a manner that eliminates the need for multiple stages, complicated preprocessing, landmark detection, or feature extraction.

The research contributions of this work are as follows:

- A highly accurate artificial intelligence system for the diagnosis of DDH is developed based on radiographic X-ray images of the pelvis. Such a product has the potential to support clinical decision-making and reduce errors and overhead.
- Numerous X-ray images of DDH patients are collected. This dataset will be made publicly available, which will benefit research in the area and the education/training of medical students.
- The performance of thirteen deep transfer learning models is thoroughly evaluated and compared using various setups and metrics that can reveal any strengths/shortcomings.

The remainder of this paper is organized as follows. The methods section describes the general steps taken to develop the DDH diagnosis system, the dataset, deep learning models, the experimental setup and performance metrics. The results are described and discussed in the results section. In the final section, we present our conclusions.

## Methods

**Fig. 2:**
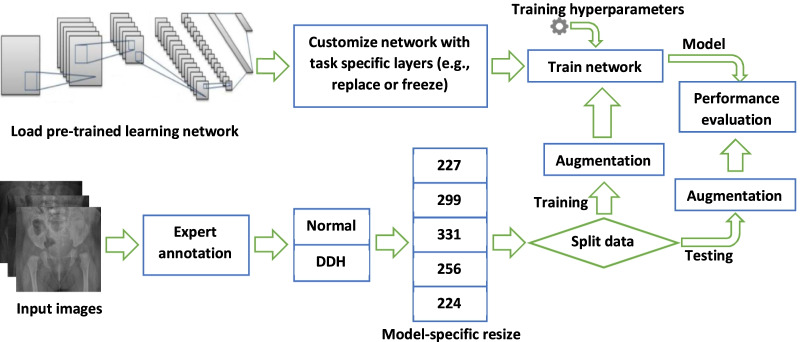
A graphical representation of the steps used in this work

The general steps used to build and test the DDH detection models are shown in Fig. 2. The approach used in this work does not require any landmark detection, nor does it rely on an explicit feature extraction. Moreover, no angle measurements are needed. All of these aspects are automatically handled by the intricacies of the deep learning model layers and operations. The next few subsections explain each part in detail.

### Dataset

The pelvic X-ray images, in an anteroposterior (AP) view, were collected locally by the authors at King Abdullah University Hospital, Jordan University of Science and Technology, Irbid, Jordan and at Alsafa specialized hospital, Jarash, Jordan. The dataset used in this work included 354 subjects (120 DDH, 234 normal) with a mean age of $4.5 \pm 0.83$ months and a maximum of 7 months. The images were taken as part of a standard diagnostics/screening procedure for infants to check for DDH. The images were ordered by the specialists at KAUH and Alsafa Hospital and processed by the designated radiologists at the corresponding hospital. The diagnosis (normal or DDH) was determined by three specialists upon X-ray inspection.

Only one image per subject was included in the dataset in one of the two classes (i.e., DDH or normal). The original images were in a high resolution JPEG format (i.e., larger than 2000$\times$2000 pixels). The images were later resized to match the specific deep learning model requirements. Aside from cropping to remove the irrelevant black parts of the image, no other preprocessing operations were performed.

### Deep learning models

Instead of building the CNNs per application from scratch, transfer learning allows the use of highly capable pretrained networks. The main operational premise is that training a large model on a very large and diverse dataset will serve as a template for specific applications. Initial layers will learn generic features (e.g., color), whereas the later layers will serve the specific application. This approach has been successfully used in many applications in the literature [26].

In this work, thirteen deep learning CNN pretrained models were used individually to detect DDH in hip X-ray images. These were DarkNet-53 [27], DenseNet-201 [28], EfficientNet-b0 [29], GoogLeNet [30], Inceptionv3 [31], Inception-ResNet, MobileNetv2, ResNet-(18, 50, and 101) [32], ShuffleNet [33], SqueezeNet [34] and Xception [35]. The models differ in their input size, width of the network and the number of layers (i.e., depth). Moreover, some models introduced changes for computational efficiency (e.g., residual networks). All models were pretrained using the ImageNet dataset [36].

### Experimental setup

The hyperparameters for all the models were set as follows: The minimum batch size, which controls the computational efficiency, was set to 16. Depending on the model, larger values may not be possible due to the large memory requirements. The maximum number of epochs was set to 50 to allow the learning process to peak while avoiding unnecessarily lengthening the training time (i.e., training with a flat loss curve in later epochs). The learning rate was set to 0.0003. The stochastic gradient descent with momentum (SGDM) optimization algorithm was used as the solver for network training. It is widely used for training due to it fast convergence [37].

Two methods for splitting the data into training/testing were evaluated: 70/30 and 90/10. This will test the models’ ability to learn if they are fed more data, and give more insight into the learning process (e.g., overfitting or underfitting).

**Fig. 3:**
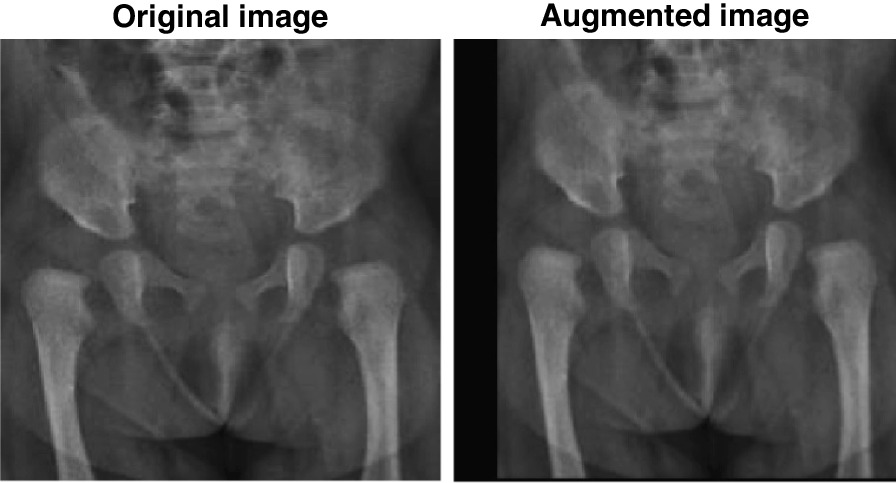
The effect of image augmentation

Input images were augmented by performing random x-axis and y-axis translations (i.e., moving the image along those axes) using the pixel range [− 30 30] and a random scaling using the scale range [0.9 1.1]. An augmentation has been found to improve the learning process by preventing overfitting (i.e., optimizing the model for specific image details) [38]. This step does not increase the size of the dataset because the augmented dataset replaces the original dataset. Hence, the results are not artificially improved by duplication. Figure 3 shows the effect of image augmentation.

The deep learning models were customized, trained, and evaluated using MATLAB R2021a software running on an HP OMEN 30L desktop GT13 with 64 GB RAM, NVIDIA GeForce RTX^TM^ 3080 GPU, Intel Core^TM^ i7-10700K CPU @ 3.80GHz, and 1TB SSD.

### Performance evaluation metrics

The performance was evaluated using the metrics in Eqs. 1–5, where TP is true positive, FN is false negative, FP is false positive and FN is false negative. The recall (i.e., sensitivity or true positive rate) measures the ability to identify DDH X-rays as positive. On the other hand, specificity (i.e., true negative rate) measures how many normal X-rays are correctly identified as such (i.e., negative or normal). An overly sensitive system will identify a high percentage of positive cases, which may be at the expense of additional false positives. Thus, precision is required to report the percentage of true positive (i.e., DDH) images as a percentage of all the reported positive images, including the false images. Accuracy determines the ratio of correctly identified positive and negative cases to the total number of images. In the case of imbalanced datasets with large disparities in the number of images in each class, the F1 score provides a good indicator of accuracy [39]. In addition, the training and validation times were reported.

1 $$\begin{aligned} \text {Recall}= & {} \frac{\text {TP}}{\text {TP}+\text {FN}} \end{aligned}$$

2 $$\begin{aligned} \text {Specificity}= & {} \frac{\text {TN}}{\text {TN}+\text {FP}} \end{aligned}$$

3 $$\begin{aligned} \text {Precision}= & {} \frac{\text {TP}}{\text {TP}+\text {FP}} \end{aligned}$$

4 $$\begin{aligned} \text {F1 score}= & {} 2\times \frac{\text {Precision}\times \text {Recall}}{\text {Precision}+ \text {Recall}} \end{aligned}$$

5 $$\begin{aligned} \text {Accuracy}= & {} \frac{\text {TP}+\text {TN}}{\text {TP}+\text {FP}+\text {TN}+\text {FN}} \end{aligned}$$

The receiver operating characteristic (ROC) curve and the corresponding area under the curve (AUC) were also used in the performance evaluation. The ROC and AUC are typically used to show the compromise between the false positive rate (i.e., 1 - specificity) versus the true positive rate (i.e., recall). In other words, it investigates the effect of varying the threshold for accepting cases as positive. A good model will have high recall values for high values of specificity (i.e., low false positive rate) and maintain a high true positive rate throughout. Thus, the higher the ROC curve and the more to the left it is, the better the performance. This is reflected in the value of the AUC.

## Results

The objective of the experiments was to evaluate the ability of the customized and retrained models to correctly identify X-ray images that show DDH. In addition, the time required for training and validation was reported.

**Fig. 4:**
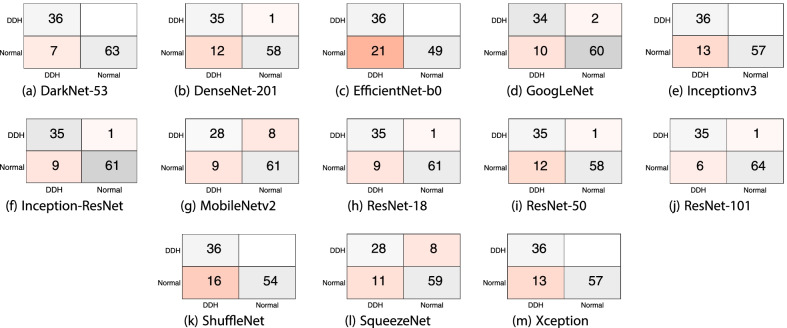
Sample confusion matrices for all the algorithms using 70/30 data split

**Fig. 5:**
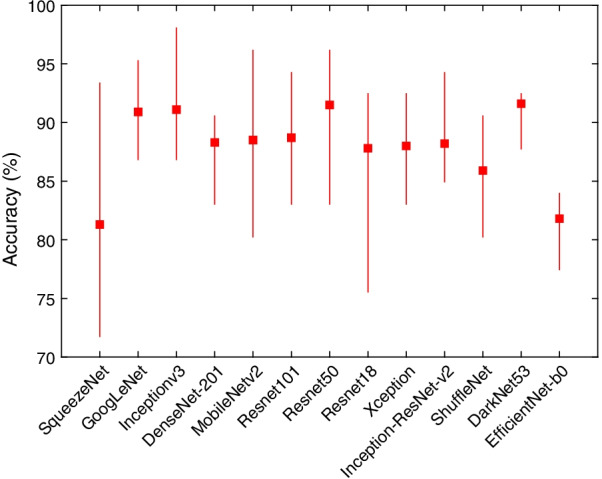
The mean, minimum, and maximum accuracy for all the algorithms over 10 randomized runs using 70/30 data split

**Fig. 6:**
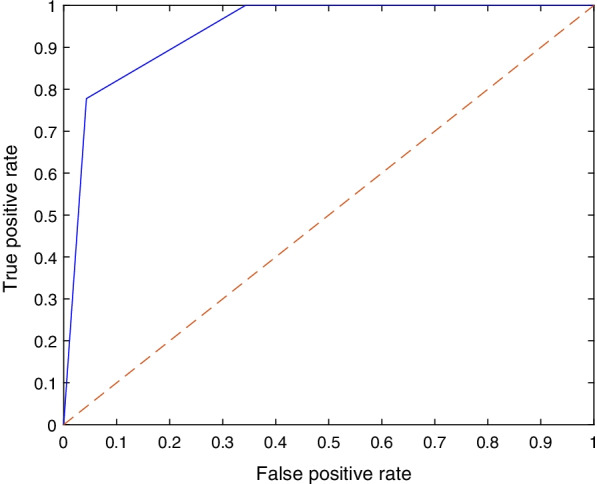
A sample ROC curve for Inceptionv3 using 70/30 data split. The AUC was 93.57%

Table 1 shows the mean F1 score, precision, recall and specificity for each deep learning model and the 70/30 data split. Most models, aside from SqueezeNet and EfficientNet-b0, achieved comparable F1 scores, with DarkNet-53 achieving the highest value (88.9%). All the models exhibited high sensitivity to the DDH class and achieved very high values, with Inceptionv3 correctly identifying all cases. The table clearly shows that the source of the errors is the false positives (i.e., identifying normal images as DDH). However, the SqueezeNet model was consistent over the two classes (DDH and normal) in contrast to the other models or even EfficientNet-b0. The sample confusion matrices in Fig. 4 corroborate these observations. The mean, minimum and maximum accuracies for all the algorithms using a 70/30 data split are shown in Fig. 5. The figure gives an indication of the performance fluctuation of the various models with different random choices of images for training and testing. The Darknet53 model produced the least variance, and SqueezeNet produced the most variance. Moreover, as the number of layers (i.e., depth) is increased in the Resnet models, the fluctuation in the accuracy with different random choices decreases. Figure 6 shows a sample ROC curve for the Inceptionv3 model, as one of the best performing models, using a 70/30 data split. The ROC curve displays excellent performance, although there is obviously room for improvement, and a larger dataset will help generate a smoother curve. The AUC value was 93.57%.

**Table 1:** The mean F1 score, precision, recall, and specificity for each deep learning model using 70/30 data split over 10 randomized runs

Deep learning model	F1 score	Precision	Recall	Specificity
SqueezeNet	76.6%	71.0%	86.4%	78.7%
GoogLeNet	87.8%	82.3%	94.4%	89.1%
Inceptionv3	88.6%	79.8%	100.0%	86.6%
DenseNet-201	85.2%	75.1%	98.6%	83.0%
MobileNetv2	85.3%	76.7%	96.7%	84.3%
Resnet101	85.7%	76.0%	98.6%	83.6%
Resnet50	88.8%	81.9%	97.5%	88.4%
Resnet18	84.7%	75.3%	97.2%	83.0%
Xception	84.9%	74.7%	98.6%	82.6%
Inception-ResNet-v2	84.7%	75.9%	96.1%	84.1%
ShuffleNet	82.7%	71.7%	98.1%	79.7%
DarkNet-53	88.9%	80.9%	98.9%	87.9%
EfficientNet-b0	78.5%	65.5%	98.1%	73.4%

**Fig. 7:**
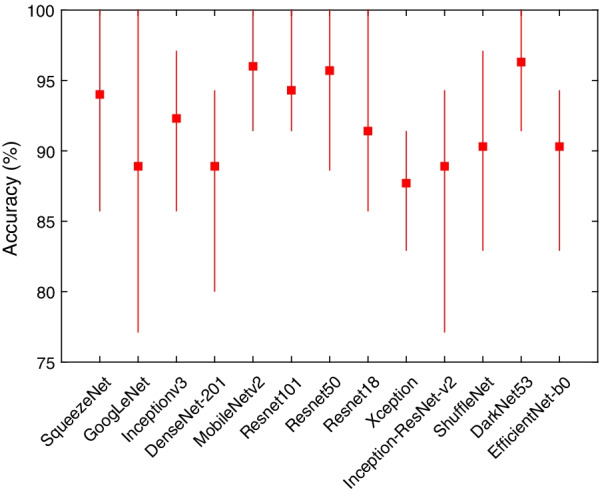
The mean, minimum, and maximum accuracy for all the algorithms over 10 randomized runs using 90/10 data split

**Fig. 8:**
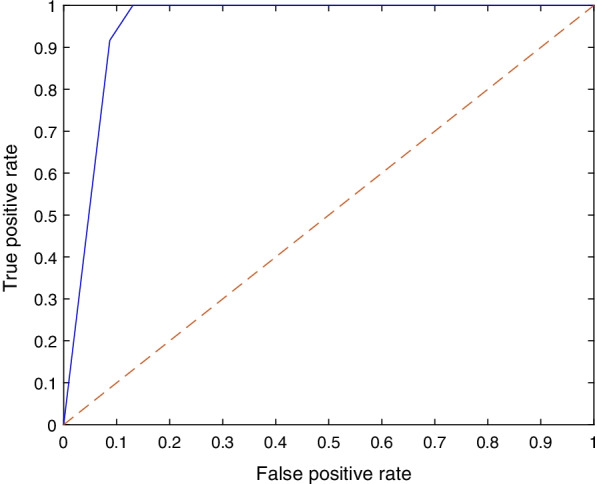
A sample ROC curve for DarkNet-53 using 90/10 data split. The AUC was 95.11%

Although the number of subjects and images used in this work is larger than most of the related studies in the literature, the deep learning models require large datasets and achieve better performance with an increased number of training samples. To evaluate the performance of the models when they are fed with more training data, further experiments were performed with a 90/10 data split. Table 2 shows the mean F1 score, precision, recall and specificity for each deep learning model and the 90/10 data split. All models achieved near perfect sensitivity (i.e., recall) by correctly identifying DDH images. The SqueezeNet model seemed to benefit the most from the increased number of training images, with the performance metrics greatly improving over the ones reported for the 70/30 data split (see Table 1) and they exhibited less fluctuation (see Fig. 7). Moreover, the DarkNet-53 model remained in the lead with a 95% F1 score. Figure 8 shows a sample ROC curve for the DarkNet-53 model, as the best performing model, using a 90/10 data split. The ROC curve displays better performance than the curve in Fig. 6. The smoothness of the curve is not visible due to having most of the recall values close to or equal to 1. The AUC value was 95.1%.

**Fig. 9:**
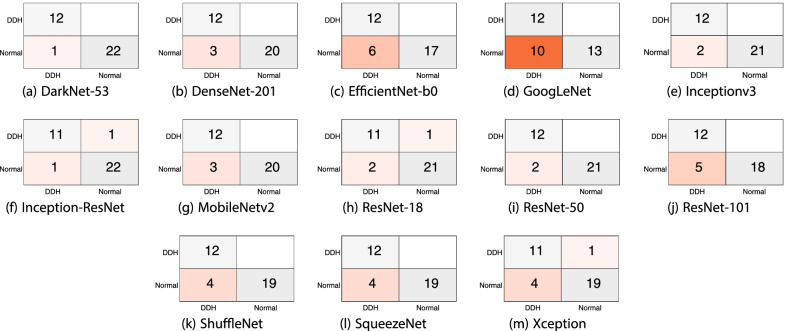
Sample confusion matrices for all the algorithms using 90/10 data split

**Fig. 10:**
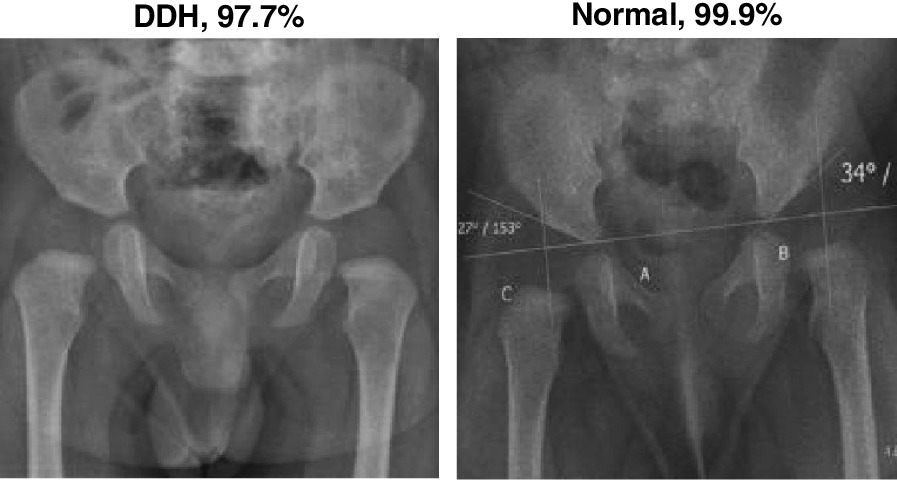
A sample of wrongly classified X-ray images

**Fig. 11:**
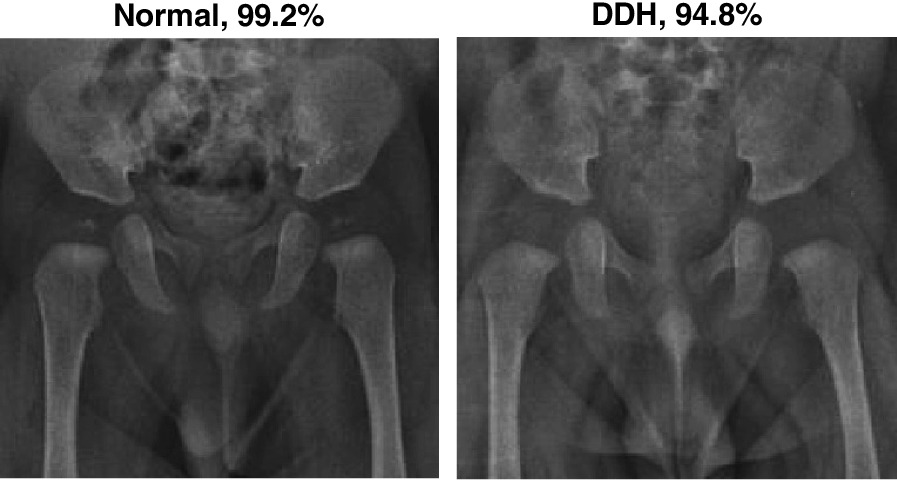
A sample of correctly classified X-ray images

The confusion matrices in Fig. 9 show a near perfect detection of DDH cases, with the false positives as the cause of errors. However, such errors may be tolerable in a screening system. Although having both high precision and recall is desirable, in a dependable system, it is better to report false positives that can be detected with further tests and evaluation by specialists than to miss reporting positive cases that will worsen with time. Figures 10 and 11 show samples of the wrongly and correctly classified pelvic X-ray images, respectively, along with the detection probabilities.

**Fig. 12:**
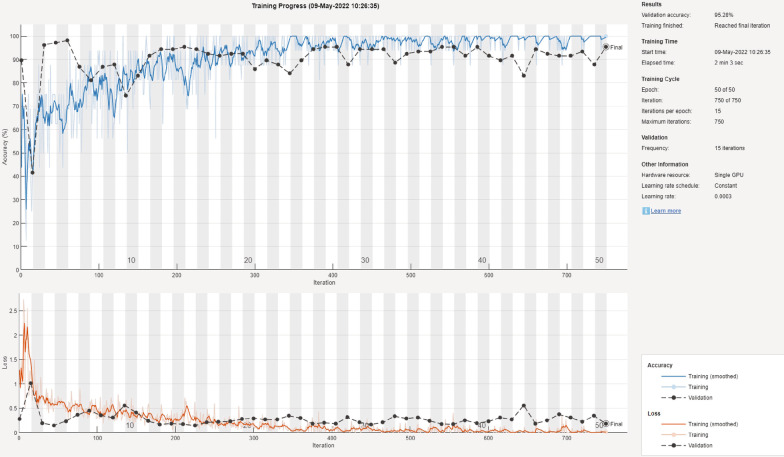
Training and validation progress for the GoogLeNet model using 70/30 data split

**Fig. 13:**
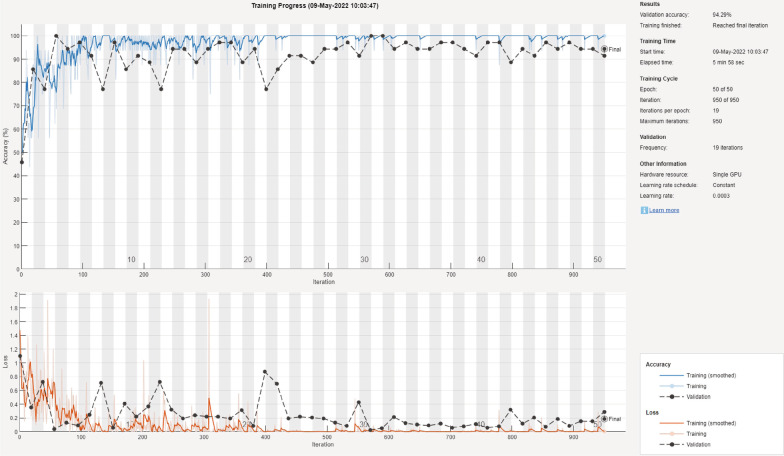
Training and validation progress for the DarkNet-53 model using 90/10 data split

It is important to report the training and validation behavior of the deep learning models to expose models that overfit or underfit the data. For two of the best performing models, Fig. 12 shows a sample of the training and validation progress for the GoogLeNet model using 70/30 data split and Fig. 13 shows a sample of the training and validation progress for the DarkNet-53 model using 90/10 data split. Both figures show a stable learning process with a decreasing loss and no apparent overfitting/underfitting.

**Table 2:** The mean F-score, precision, recall, and specificity for each deep learning model using 90/10 data split

Deep learning model	F1 score	Precision	Recall	Specificity
SqueezeNet	92.3%	86.2%	100.0%	90.9%
GoogLeNet	86.5%	79.6%	96.7%	84.8%
Inceptionv3	90.0%	82.1%	100.0%	88.3%
DenseNet-201	86.2%	76.6%	99.2%	83.5%
MobileNetv2	94.6%	90.1%	100.0%	93.9%
ShuffleNet	82.9%	71.6%	98.8%	79.6%
Resnet101	92.5%	86.3%	100.0%	91.3%
Resnet50	94.4%	89.8%	100.0%	93.5%
Resnet18	89.1%	81.4%	99.2%	87.4%
Xception	83.9%	76.4%	93.3%	84.8%
Inception-ResNet-v2	86.1%	76.4%	99.2%	83.5%
ShuffleNet	87.7%	78.9%	99.2%	85.7%
DarkNet-53	95.0%	90.6%	100.0%	94.3%
EfficientNet-b0	87.7%	78.3%	100.0%	85.2%

Table 3 shows the training and validation times for all the models using the 70/30 and 90/10 data splits. The times increase linearly with a 20% increase in the training data. The fastest model was SqueezeNet with less than a minute and a half for either training data size. The DarkNet-53 model requires a reasonable amount of training time (317.6 and 362 seconds) given that it achieved the highest performance. These times do not generally affect the usability of the model, as testing times were on the order of milliseconds per image and the training is done once and offline with respect to the deployment.

**Table 3:** The mean training and validation time for each deep learning model (in seconds)

Data split	70/30	90/10
Deep learning model		
SqueezeNet	75.9	84.2
GoogLeNet	129.8	152.8
Inceptionv3	356.7	413.5
DenseNet-201	1261.3	1451.6
MobileNetv2	586.7	674.6
ResNet-101	392.6	454.9
ResNet-50	185.7	205.8
ResNet-18	76.82	82.0
Xception	1431.7	1629.0
Inception-ResNet-v2	1332.0	1363.7
ShuffleNet	443.9	492.7
DarkNet-53	317.6	362.0
EfficientNet-b0	983.1	1331.5

In regards to the related literature and to our knowledge, the problem of direct diagnoses of DDH from X-ray images has received little attention. Table 4 shows some of the related results in the literature. In Sahin et al. [24], comparing means is not a valid accuracy metric, as it does not compare individual measurements to the corresponding gold standard. Liu et al. [25] report a 94.68% F1 score, however, this is done for the heavy dislocation case based on their approach for measuring the AA index, and it is not clear what gold standard did they compare to given that they compared their results to a doctor with/without the aid of a computer. Moreover, the work in this paper takes a direct approach to DDH detection, which avoids the compounded errors in landmark detection and manual/automatic measurements.

The present study has some limitations. First, the size of the dataset needs to be much larger to realize the full potential of deep learning AI. Second, different medical norms may affect the diagnosis; for example, some doctors consider the X-ray to be DDH positive if the AA angle is greater than 30°, while others may require the angle to be less than 25°for normal diagnosis. In addition, the age of the child being examined may play a role in the diagnosis (e.g., an AA angle may be acceptable for a younger age group but not for older ones). Third, full angle measurements were not included or available for the dataset. Such data enable updates to the diagnosis to match different standards. Moreover, it opens the door for studies to calculate the various involved angles (e.g., AA angle) using deep learning regression.

For future and more robust studies, it is possible to develop custom classification models using different architectures and diverse/larger datasets. Moreover, the development of ensemble deep learning models for DDH classification is another avenue for research. In addition, exhaustive evaluation with different augmentation operations, preprocessing techniques and hyperparameters is preferred. Regarding deployment, the extra effort of wrapping deep learning models into smartphone applications should be worthwhile.

**Table 4:** A comparison to the related results in the literature

Study	Dataset	Objective	Performance
Al-Bashir et al. [22]	16 subjects/images	AA measurement	85% accuracy
Xu et al. [21]	9369 images (0.1–14 years)	Landmark detection, age of femoral head	4.46 average pixel error 89.3% accuracy
Sahin et al. [24]	157 X-ray images	AA measurement	–
Liu et al. [25]	10,000 pelvis x-ray images	Landmark detection	F1: 94.68% (heavy dislocation)
This work	354 subjects/images	DDH vs Normal diagnosis	F1: 95.0%

## Conclusions

Developmental dysplasia of the hip (DDH) is a common disorder among newborns, and increased screening and radiographic imaging have revealed an even higher prevalence than previously thought. Early detection of DDH has been shown to drastically reduce the bracing time and the need for surgery, and prevents further painful long-term complications. However, an accurate diagnosis requires specialist knowledge of the development of the pelvis, specific measurements and a determination of the relative position of several key landmarks, which is an error-prone and time-consuming process.

In this work, we collected X-ray images of DDH patients, utilized recent advances in deep convolutional neural networks and applied transfer learning to the problem of DDH diagnosis. Such an approach has the potential to achieve high diagnosis accuracy with little overhead being incurred to the specialists. Moreover, it does not require explicit measurements, manual preprocessing, or compounded errors. Future work will focus on improving the system by expanding the dataset and applying incremental learning approaches to evolve the application during deployment. Moreover, we will consider 3D tests for the DDH measurements as they are becoming common in identifying certain cases of DDH [4] and the application of T önnis and the International Hip Dysplasia Institute (IHDI) standard classifications for DDH severity. In addition, the development of ensemble deep learning models for DDH classification is another avenue for research. Furthermore, an exhaustive evaluation with different augmentation operations, preprocessing techniques, and hyperparameters is preferred. Regarding deployment, the extra effort of wrapping deep learning models into smartphone applications should result in tangible benefits.

## Data Availability

The dataset used in this work is available privately at https://data.mendeley.com/datasets/jf3pv98m9g/1 and will be made available publicly at Mendeley data repository upon the acceptance of this article at www.doi.org/10.17632/jf3pv98m9g.1. Moreover, the data are available from the corresponding author upon reasonable request.

## Abbreviations

AA: Acetabular angle
AP: Anteroposterior
AUC: Area under the curve
DDH: Developmental dysplasia of the Hip
CNN: Convolutional neural networks
GPU: Graphical processing units
GMDH: Group method of data handling
HRNet: High resolution network
IHDI: International Hip Dysplasia Institute
Mask-RCNN: Mask-region based convolutional neural networks
PN-UNet: Pyramid nonlocal UNet
ReLU: Rectified linear unit
ROC: Receiver operating characteristic
SGDM: Stochastic gradient descent with momentum
